# Evaluation and Source Apportionment of Heavy Metals (HMs) in Sewage Sludge of Municipal Wastewater Treatment Plants (WWTPs) in Shanxi, China

**DOI:** 10.3390/ijerph121215022

**Published:** 2015-12-11

**Authors:** Baoling Duan, Fenwu Liu, Wuping Zhang, Haixia Zheng, Qiang Zhang, Xiaomei Li, Yushan Bu

**Affiliations:** 1 College of Resources and Environment, Shanxi Agricultural University, Taigu, Shanxi 030801, China; sxnddbl@163.com (B.D.); lfwlfw2008@sina.com (F.L.); zwping@126.com (W.Z.); zhenghaixia81@126.com (H.Z.); 18334759065@163.com (Q.Z.); 2 College of Forestry, Shanxi Agricultural University, Taigu, Shanxi 030801, China; 3 Alberta Innovates—Energy & Environment Solutions, Edmonton, AB T5J3G2, Canada

**Keywords:** sewage sludge, heavy metals, geoaccumulation index, classes of pollution, principal component analysis, source apportionment, Shanxi

## Abstract

Heavy metals (HMs) in sewage sludge have become the crucial limiting factors for land use application. Samples were collected and analyzed from 32 waste water treatment plants (WWTPs) in the Shanxi Province, China. HM levels in sewage sludge were assessed. The multivariate statistical method principal component analysis (PCA) was applied to identify the sources of HMs in sewage sludge. HM pollution classes by geochemical accumulation index *I*_geo_ and correlation analyses between HMs were also conducted. HMs were arranged in the following decreasing order of mean concentration: Zn > Cu > Cr > Pb > As > Hg > Cd; the maximum concentrations of all HMs were within the limit of maximum content permitted by Chinese discharge standard. *I*_geo_ classes of HMs pollution in order from most polluted to least were: Cu and Hg pollution were the highest; Cd and Cr pollution were moderate; Zn, As and Pb pollution were the least. Sources of HM contamination in sewage sludge were identified as three components. The primary contaminant source accounting for 35.7% of the total variance was identified as smelting industry, coking plant and traffic sources; the second source accounting for 29.0% of the total variance was distinguished as household and water supply pollution; the smallest of the three sources accounting for 16.2% of the total variance was defined as special industries such as leather tanning, textile manufacturing and chemical processing industries. Source apportionment of HMs in sewage sludge can control HM contamination through suggesting improvements in government policies and industrial processes.

## 1. Introduction

Many wastewater treatment plants (WWTPs) were built during the past decades in China [1,2]. In the Shanxi province, with a population of 35 million, more than 158 WWPTs were built in the past ten years. More and more sewage sludge will be produced with the increasing population and number of WWTPs. During 2010, China produced approximately 2.66 million tons of sewage sludge [3]. In consequence, sewage sludge has become an imminent environmental problem in China [4,5].

Traditional utilization of sewage sludge is land application as soil fertilizer or amendment to promote crop production because of its organic matter, N, P, K and other plant nutrients in sewage sludge [6,7,8,9]. Europe has utilized over 50% of sewage sludge in agricultural soils [10]. Poland alone applied 0.116 million tons of sewage sludge (dry) in agriculture in 2011 [11]. However, this practice may lead to heavy metal (HM) accumulation and contamination in agricultural soils [12,13]. Excessive cumulative HMs in agricultural soil may lead to potential human health risk when HMs migrate from soil to other ecosystems such as underground water or human food sources [14,15]. Therefore, HM content is a crucial limiting factor for application of sewage sludge in agricultural land in many countries [16,17]. Studies on HM in sewage sludge generally focus on content distribution, trend, removal technology and risk assessment [1,2,3]. Few surveys study the sources apportionment of HMs in sewage sludge, most research on sources of HMs in soil or lake sediment [15,17]. Unmix receptor model, factor analysis, positive matrix factorization and PCA are used to identify source apportionment of HMs [15,17,18]. Storm water, drainage and roof leakage water, industry and traffic as well as house hold all contribute to the supply of HMs [19,20]. Identifying the sources apportionment of HMs in sewage sludge is vitally important for targeted reduction of HMs in WWTPs [21,22].

Shanxi province is the biggest coal base in China, and it is abundant of other metallic mineral resources like iron mines [23,24]. In this province, over 82% of the energy is consumed in its five largest industries including coal, coke, metallurgy, electricity and chemical production [24,25]. These industries affecting HM content in sewage sludge are typical in China. However, little literature exists on contamination and source apportionment of HMs in sewage sludge about this important province.

The purpose of this study was to assess the HMs levels and identify their sources in sewage sludge generated from WWTPs across studying area. Three main objectives are (a) to identify the concentration of HMs in sewage sludge sampled from municipal WWTPs; (b) to assess the class of HM contamination in different samples; and (c) to identify source apportionment of HMs in sewage sludge across Shanxi province, China.

## 2. Materials and Methods

### 2.1. Sampling and Chemical Analysis

**Figure 1:**
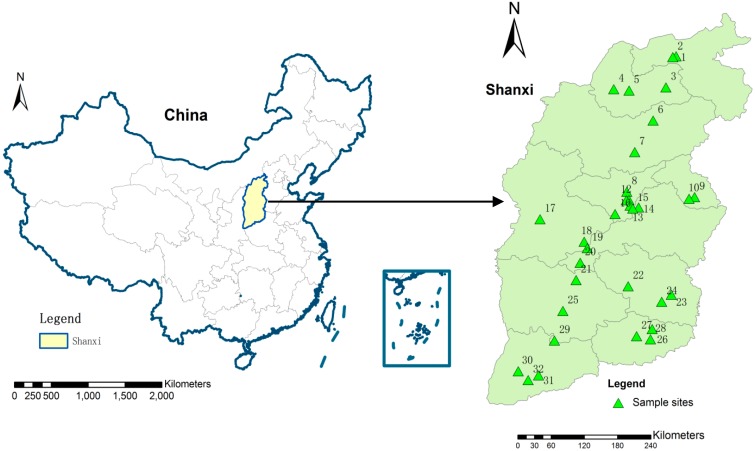
Sampling sites of wastewater treatment plants (WWPTs) locations in Shanxi province, China.

Secondary dewatered sludge was collected from 32 municipal WWTPs across Shanxi Province, China, as indicated in Figure 1. To enhance samples representative, subsamples were collected from four different sites in the storage pile at each WWTP. Four subsamples from one WWTP mixed together as one sample. Coordinate system locations of sewage sludge samples were recorded using a GPS receiver. This allowed selecting representative WWTP sites and locating heavy industry plants.

Collected samples were dried in clean environment in air at room temperature, then sieved through to a mesh pore size of 0.14 mm and stored in jars at room temperature. Dry sludge samples were weighted and digested with HNO_3_ using a microwave digestion system (Mars 5, CEM, America) by United States Environmental Protection Agency Method 3051B. Cu, Zn, Cr and Pb were analyzed by atomic absorption spectrophotometer (WARIAN-AA-240, WARIAN, America), Cd was analyzed by graphite furnace atomic absorption spectrophotometer (TAS-990AFG, PERSEE, Beijing, China), and As and Hg were analyzed by atomic fluorescence spectrometer (AFS-230E, Haiguang, Beijing, China). To control quality, certified reference sludge samples (RTC-CRM055, TMRM, China) and national standards of China (GB/T 15555.2-1995, GB/T 15555.2-1995, GB/T 15555.6-1995, GB/T 15555.2-1995, GB/T 17141-1997, GB/T 22105.2-2008, GB/T22105.1-2008) were used. The accuracy and precision of analysis were checked by testing certified reference material six times, results are shown in Table 1. Blank samples were performed 11 times to determine detection limit (Table 1). When each batch of samples tested, two blank samples and reference samples were detected at the same time. Triplicate samples were determined, and the mean value of result was the final concentration of HMs.

**Table 1:** Analytical accuracy, precision, recover and detection limit.

Metals	Accuracy (%)	Precision (%)	Recover (%)	Detection Limit (mg/kg)
Cu	2.1	–2.0	94.5	0.9
Zn	1.3	–2.1	96.6	1.0
As	1.8	–1.9	97.0	0.013
Hg	5.6	–7.6	92.2	0.003
Pb	3.5	–5.1	93.2	0.5
Cd	2.8	6.4	106.1	0.06
Cr	1.8	–2.9	95.4	4.2

### 2.2. Assessment of HM Contamination

The geoaccumulation index (*I*_geo_) was developed and used for assessing the pollution of HMs in bottom sediments since the 1960s [26,27]. It evaluates the pollution level of a particular HM in comparing with its geochemical background concentration [28]. *I*_geo_ is defined as the following equation [26].

$$I_{\text{geo}}={\text{Log}}_{2}\left(\frac{{\text{C}}_{\text{n}}}{1.5{\text{B}}_{\text{n}}}\right)$$

where C_n_ is the concentration of HM (n) examined in sediment samples and B_n_ is geochemical background concentration of this HM (n). The factor of 1.5 is the background matrix correction factor due to lithospheric effects. Geochemical background concentration is generally represented by global average shale data [29,30,31,32]. *I*_geo_ was classified into seven grades by Muller in 1981 as shown in Table 2 [28]. This paper used *I*_geo_ to classify the HM contamination in sewage sludge.

**Table 2:** Geo-accumulation index (*I*_geo_) and classes used for evaluation heavy metal (HM) contamination [26].

*I*_geo_	Class	Level of Contamination
≤0	0	practically unpolluted
0–1	1	unpolluted to moderately polluted
1–2	2	moderatelypolluted
2–3	3	moderately toheavily polluted
3–4	4	heavily polluted
4–5	5	heavily to extremely polluted
>5	6	extremely polluted

### 2.3. Data Analysis

Contents and relationships between each other and the source apportionment of HMs in sewage sludge were analyzed. Correlations between HMs were identified by Pearson correlation analysis. Principal component analysis (PCA), a multivariate statistical technique, was performed by SPSS19.0 to identify source apportionment of HMs. It can make a distinction between lithogenic sources and anthropogenic sources of pollution [33,34,35].

PCA is proposed by Hotelling in 1933 [11]. It enables a reduction in data and depicts a given multidimensional system through a small number of new variables [34,35]. There is a brief summary of PCA given here. X is a n × p matrix (*n* = number of observations, *p* = number of variables). PCA synthesizes the observing variables into new variables as *F*_1_ to *F*_p_, and the numbers of new variables are the same as before, whichcan be represented as the following:

$$F_{1}=a_{11}x_{1}+a_{12}x_{2}+\cdots +a_{1p}x_{p}$$

$$F_{2}=a_{21}x_{1}+a_{22}x_{2}+\cdots +a_{2p}x_{p}$$

$$F_{p}=a_{p1}x_{1}+a_{p2}x_{2}+\cdots +a_{pp}x_{p}$$

It can be briefly performed by the following equations:

$$\begin{array}{c} F_{j}=a_{j1}x_{1}+a_{j2}x_{2}+\cdots +a_{jp}x_{p}\\ j\;=\;1,\;2,\;\ldots ,\text{ p} \end{array}$$

j is the number of observing variables; *F*_j_ is the new variable as the jth factor, and the variance of *F*_j-1_ is bigger than that of *F*_j,_ and *F*_j-1_ is uncorrelated with *F*_j_; x_p_ is the pth observing variable; a_jp_ is the loading of the pth variables in the jth factor.

In the evaluation, Varimax rotation is often applied to maximize the component loading sand eliminate the invalid component [36]. The eigenvalue of relevant component taken into consideration should be higher than one according to the Kaiser criterion [37]. Cumulative loadings of principal factors should account for approximately 75% according to Morrison [38]. Through mapping multidimensional data into lower dimensions, PCA can efficiently identify relationships between HMs and interpret source apportionment of HMs [39,40,41]. Assessment of contamination level by *I*_geo_ and identification of correlation between HMs will be performed in order to assist source apportionment of HMs. Related metals which load higher in one component are expected more from the same source [39].

## 3. Results and Discussion

### 3.1. Level of HMs in 32 Sewage Sludge Samples

The mean concentrations of Zn, Cu, Cr, Pb, As, Hg and Cd were in the following decreasing order: Zn > Cu > Cr > Pb > As > Hg > Cd, and mean value of each HMs individually was 281.31 mg/kg, 162.59 mg/kg, 152.26 mg/kg, 39.56 mg/kg, 14.83 mg/kg, 2.09 mg/kg and 1.97 mg/kg in Table 3. Zn content was the most, while the Cd content was the least.

Because samples were from 32 WWTPs located in different regions, HM concentrations in sewage sludge all varied greatly. The concentration of Zn, Cr, Cu, Pb, Cd, As and Hg varied individually from 52.27 to 1613.50 mg/kg, 33.29 to 665.31 mg/kg, 56.15 to 520.53 mg/kg, 13.43 to 89.08 mg/kg, 0.33 to 17.23 mg/kg, 7.87 to 29.15 mg/kg and 0.15 to 7.99 mg/kg. It can be sorted by standard deviation in the following decreasing order: Zn > Cr > Cu > Pb > Cd > As > Hg (Table 3). The values of standard deviation of Zn, Cr and Cu were much greater than that of Pb, Cd, As and Hg. This can be attributed to the variable anthropogenic sources in studying area [42].

**Table 3:** Statistical analysis of heavy metal (HM) concentrations (mg/kg).

Metals	Minimum	Maximum	Mean	Range	Std. dev.	GB18918-2002 ^a^	
						pH≥6.5	pH<6.5
Cu	56.15	520.53	162.59	464.38	105.80	1500	800
Zn	52.27	1613.50	281.31	1561.23	336.66	3000	2000
As	7.87	29.15	14.83	21.27	4.96	75	75
Hg	0.15	7.99	2.09	7.83	1.95	15	5
Pb	13.43	89.08	39.56	75.66	19.81	1000	300
Cd	0.33	17.23	1.97	16.90	3.55	20	5
Cr	33.29	665.31	152.26	632.02	131.65	1000	600

**^a^**Chinese discharge standard of pollutants for Municipal WWTP for agricultural use (GB 18918-2002).

Comparing with the threshold values of Chinese Discharge Standard of Pollutants for Municipal WWTP (GB 18918-2002) for agricultural use in Table 3, the maximum concentrations of HMs were all within the content limits permitted by discharge standard.

Comparing HM contents in studying area with other regions in China (Table 4) [3,4,43], Cu, Zn, Hg, Pb, Cd and Cr were relatively lower and only As was practically equal to the value in north of China. The result indicated that HM pollution of sewage sludge in Shanxi was not as severe as other regions in China. Because of the wider range of sampling compared to other studies, ranges of HM concentrations in this study are all higher than other surveys on Shanxi [1,2,3].

**Table 4:** HM contents in different regions of China (mg/kg).

Region	Cu	Zn	As	Hg	Pb	Cd	Cr
North	535.95	1220.88	14.20	5.81	113.11	7.33	242.71
South	530.96	1301.63	18.03	2.99	116.22	7.09	208.70
East	671.87	1446.23	15.94	3.92	140.47	7.32	209.36
West	249.25	717.55	20.40	3.80	80.63	7.55	161.40
Mean values of Shanxi	162.59	281.31	14.83	2.09	39.56	1.97	152.26

### 3.2. Contamination of HMs in Sewage Sludge, Shanxi

Contaminations of HMs in sewage sludge of 32 WWTPs were classified from class 0 to 5 according to *I*_geo_ (Table 5). Results depicted that Cu and Hg pollution were the highest; Cd and Cr pollution were moderately; Zn, As and Pb pollution were the least. Overall, HM pollution in sewage sludge in Shanxi was generally not significant. To better understand HM contamination in each site, type of process were presented in Table 5.

Although HM pollution was overall not serious, it was notable in some samples. Hg pollution class varied mostly ranging from class 0 to 4. For Hg, 40.63% samples were in class 0 and class 1; 25% samples were in class 2 and 34.37% samples were in class 3 to 4. Cu was another serious contaminated metal: 15% samples were in class 0 and 1, while the others were all in class 2 to 3. It was noting that contamination of Cu and Hg generally synchronously varied with the severest pollution of Cu and Hg at the same sites WWPT 8, 10, 11, 18, 25, 29, 30, 31 and 32. This phenomenon suggested that Cu and Hg might have the same anthropogenic sources.

**Table 5:** Classes of contamination by *I*_geo_ of sewage sludge for different WWTPs in Shanxi.

WWTP	Cu	Zn	As	Hg	Pb	Cd	Cr	Type of Process
1	0	2	0	1	0	1	1	CarrouselOxidationditch
2	0	2	0	1	0	1	1	CarrouselOxidationditch
3	0	2	0	0	0	0	1	Anaerobic/Anoxic/Oxic
4	0	1	1	0	0	1	2	CarrouselOxidationditch
5	0	1	0	0	0	0	2	SequencingBatchReactor
6	2	3	0	0	0	1	2	Anaerobic/Anoxic/Oxic
7	0	1	0	0	0	1	1	Anaerobic-Oxicprocess
8	2	0	0	3	1	0	1	CarrouselOxidationditch
9	1	0	0	2	0	0	0	Activatedsludgeprocess
10	2	0	0	3	1	1	1	Anaerobic/Anoxic/Oxic
11	2	0	0	3	0	1	0	Activatedsludgeprocess
12	2	0	0	2	1	1	0	Activatedsludgeprocess
13	2	0	1	2	0	0	1	Biological contact oxidation process
14	1	0	0	4	0	0	0	SequencingBatchReactor
15	2	0	0	3	0	1	3	Anaerobic/Anoxic/Oxic
16	2	0	0	3	0	5	0	Biological contact oxidation process
17	1	0	0	4	0	0	0	Biological contact oxidation process
18	2	0	0	3	0	1	0	Activatedsludgeprocess
19	2	0	0	2	1	1	0	Anaerobic/Anoxic/Oxic
20	2	0	0	2	0	0	0	Anaerobic/Anoxic/Oxic
21	0	0	0	1	0	0	0	Anaerobic-Oxicprocess
22	1	3	1	1	0	5	1	Anaerobic/Anoxic/Oxic
23	1	2	0	0	0	2	1	Biological contact oxidation process
24	1	4	1	2	1	4	1	Anaerobic/Anoxic/Oxic
25	2	0	0	4	0	2	0	Biomembrane process
26	1	2	0	0	0	2	2	CarrouselOxidationditch
27	1	0	0	0	0	0	1	Biomembrane process
28	1	2	0	0	0	1	2	Anaerobic-Oxicprocess
29	3	0	0	4	1	2	1	Biomembrane process
30	3	0	1	4	1	2	3	Anaerobic-Oxicprocess
31	2	0	0	2	1	1	0	Biomembrane process
32	3	0	0	2	1	2	1	Biomembrane process
Mean	1.38	0.78	0.16	1.81	0.28	1.22	0.91	—

Class contamination of Cd ranged from class 0 to 5; 71.88% samples were in class 0 and 1. Only one was in class 4 and only two were in class 5, the rest 18.75% were all in class 2. It indicated that most samples were free of Cd contamination, and the heavily contaminated ones as WWTP 16, 22 and 24 were polluted by special conditions in these places. The contamination of Zn: 71.88% samples were in class 0 and 1; 18.75% were in class 2 and 9.38% were in class 3 and 4. Zn had the same variation as Cd with the most seriously polluted samples in WWPT 22 and 24. This pollution may be due to the zinc smelting industry nearby WWPT 22 and 24. For Cr, 6.25% samples were classified into class 3, 15.63% were in class 2 and the rest 78.12% were all in class 0 and 1. The pollution of Cr was moderately. Pb was almost unpolluted, 84.38% samples were in class 0, and the rest were all in class 1.

As in other regions in China, HM pollution in sewage sludge was not significant [1,2,3]. However, Hg pollution was higher in this study. That might be caused by typical industries which consume many coals distributed in Shanxi [15,22].

### 3.3. Correlation between HMs

HMs in Sewage sludge often have complicated relationships between each other with their relationships primarily controlled by anthropogenic factors such as human activities, industrial distribution *etc.* [1,40] and secondarily controlled by underlying lithology [1,22]. A correlation analysis among HMs is presented in Table 6. Significant correlations were found between Cu and Hg (*r* = 0.725), Cu and Pb (*r* = 0.658) and Hg and Pb (*r* = 0.648) at *p* = 0.01 level. Meanwhile, Zn and As (*r* = 0.467), Zn and Cd (*r* = 0.491) and As and Cd (*r* = 0.511) also have significant correlation between each other at the 0.01 level. The significant correlations between Cu-Hg-Pb and Zn-As-Cd revealed that Cu, Hg and Pb may come from the same sources, and Zn, As, and Cd may arise from same human activities [39]. The relationship between HMs is the same as other studies [15,22,27].

**Table 6:** Correlation coefficients between different HMs (*n* = 32).

Metals	Cu	Zn	As	Hg	Pb	Cd	Cr
Cu	1						
Zn	–0.256	1					
As	0.194	0.467 **	1				
Hg	0.725 **	–0.389 **	0.124	1			
Pb	0.658 **	–0.032	0.306	0.648 **	1		
Cd	–0.039	0.491 **	0.511 **	–0.002	0.148	1	
Cr	0.286	0.102	0.233	0.214	0.011	–0.066	1

Level of significance: *p<0.05, **p<0.01.

### 3.4. Potential Sources of HMs

The corresponding factors and loadings of variance were presented in Table 7 and Table 8. Three significant factors whose initial eigenvalues were greater than one had been obtained through PCA analysis, three factors explained about 80.9% of the total variance which were distinguished in the data set [43].

**Table 7:** Total variance and component matrixes for HMs in sewage sludge.

Component	Initial Eigenvalues			Extraction Sumsof Squared Loadings			Rotation Sums of Loadings		
	Total	% of Variance	Cumulative%	Total	% of Variance	Cumulative%	Total	% of Variance	Cumulative%
1	2.57	36.66	36.66	2.57	36.66	36.66	2.50	35.65	35.65
2	2.04	29.17	65.82	2.04	29.17	65.82	2.03	29.04	64.69
3	1.05	15.06	80.88	1.05	15.06	80.88	1.13	16.18	80.88

Extraction Method: Principal Component Analysis.

**Table 8:** Factor loading matrix of component matrix and rotated component matrix.

Element	Component Matrix			Rotated Component Matrix		
	Factor 1	Factor 2	Factor 3	Factor 1	Factor 2	Factor 3
Cu	0.897	–0.117	0.055	0.874	–0.051	0.237
Zn	–0.235	0.840	0.099	–0.333	0.803	0.120
As	0.347	0.786	0.088	0.241	0.796	0.232
Hg	0.888	–0.201	–0.065	0.898	–0.122	0.111
Pb	0.827	0.120	–0.326	0.861	0.219	–0.127
Cd	0.080	0.795	–0.306	0.063	0.827	–0.209
Cr	0.324	0.136	0.911	0.113	0.060	0.968

Factor 1, with the highest eigenvalue of 2.50 accounting for 35.7% of the total variance, was the most charging factor. In this factor, elements Cu (0.874), Hg (0.898) and Pb (0.861) provided with significant loadings. High correlation coefficients with Cu, Hg and Pb revealed that they might be derived from the similar pollution sources. According to high values of *I*_geo_ of Cu and Hg, factor 1 can be considered as anthropogenic component [15].

As Shanxi is a heavy-industry province with abundant mineral resources, many large industries like metallurgy and coking are distributed in the studying area. Metallurgy and coking can give rise to local contamination of Cu, Hg and Pb. When metal-processing factories are in operation, metal particles and wastewater containing Cu, Hg and Pb are generated and eventually, enter into the municipal waste water drain. At a coal-fired power plant, these metals enter the wastewater with a similar route [44]. As WWTP 14, 16, 18, 19, 25, 29 and 32 are nearby smelting and coking plants, the concentrations of Cu, Hg and Pb were higher than other samples. Level of Cu, Hg and Pb were also high for sample of WWTP 17, which was nearby a coking plant. Therefore, metallurgical industry and combustion of fossil fuels could be defined as the first sources of HM contamination in sewage sludge, and it was also certified by high relations of Cu-Hg-Pb and the greater *I*_geo_ of Cu, Hg and Pb at the same sites [33,45,46].

In addition, emission of HMs from traffic including gasoline and break lining also enters into wastewater through runoff water, suspension as particulate material and dispersal in the atmosphere away the road [47,48]. Pb pollution in sewage sludge from traffic was originated from gasoline [1,2,44]. In recent years, Pb-free gasoline has been required for use. However Pb-free does not mean that it contains no Pb. Moreover, in some places gasoline containing Pb is still in use [3,4,43]. Meanwhile, Cu can arise from traffic due to vehicle’s break lining [19,47]. The sites with high concentration of Cu and Pb like WWPT8, 10, 12, 25, 29, 30, 31 and 32 represent economic and industry-developed regions. The contaminations of Pb and Cu that happened in these places were probably due to increasing amounts of vehicular traffic.

Factor 2, accounting for 29.0% of the total variance, Zn, As and Cd, which significantly correlated with each other, had high loadings. This factor was an anthropogenic source as factor 1.

Use of galvanized pipe is the major cause of Zn contamination [1,22,47]. Distribution of galvanized pipe for the water supply had been forbidden in China since 2000, but lots of galvanized pipes are in current use [1]. When galvanized pipes were replaced and water supply pipes made of other materials were installed, Zn pollution would decrease. The level of Zn in sewage sludge from most sites was less than the national average. Only a couple showed higher levels and these were associated with nearby industries *i.e*., WWTP 6 was near a wood industry and WWTP 24 was near azinc smelting industry. Contaminations of Zn in those sites were considered as moderate to heavy. Besides this, another source of Zn contamination was car washing, which also correlated to Cd pollution in sewage sludge [47].

Low pollution classes for Cd and As indicated that origins of these two metals do not generate great deal of Cd and As. High correlation coefficient (*r* = 0.511) pointed out that they had the homologous sources. Household was the dominating source for Cd and As in sewage sludge [18,40]. Apart from household origin, inhabitants at work, in school and so on in their residences were also the source of Cd [18]. The principal source of As was the usage of arsenic containing detergent [1]. The content of As in general household detergent is approximately 3.6 × 10^−2^ mg/kg and about 1.8 mg/kg in toilet cleaner [49]. Thus, the detergents and cleaners in household are major source of As pollutants.

Factor 3, accounting for 16.2% of the total variance, only had strong positive loading only on Cr, which had poor relations with other metals. The contamination of Cr could be caused by leather tanning industries, textile manufacturing and printing and dyeing industry, as well as other industries like painting, coating and chemical engineering [50,51]. Only a few samples contamination class of Cr were in class 2 and 3. Samples of WWPT 4, 5, 6, 15, 26 and 30 were highly contaminated with Cr, and the sites of WWPT 4 and 5 are the location of the biggest tanning industry base in China; Sites of WWPT 6 and 30 are developing textile manufacturing and sites of WWPT 15 and 26 are famous of chemical engineering. This revealed that the sources of Cr contamination in Shanxi area could be attributed to the special industry as leather tanning industry, textile manufacturing or chemical engineering.

The dominant sources of HM contamination in sewage sludge were the metallurgy and coking industry, which are widely distributed in Shanxi Province. Traffic, water supply system, households and some special industries such as leather tanning industry, textile manufacturing and chemical engineering were also HM contamination sources in sewage sludge. Charging of HM pollution in sewage sludge, industry was much higher than domestic [3]. In metal and steel industries, fossil fuels have been reported to be sources of Cu, Hg and Pb pollution [38,39]. Many kinds of literature indicate that leather tanning industry and textile manufacturing can cause Cr pollution [50,51]. 9%–11% of Pb pollution was from traffic, promotion of lead-free petrol can decrease that [20]. General household detergent containing great deal of As and Cd in China will lead to great pollution in waste water [1,3]. Detergent reducing As and Cd can mitigate contamination of it in sewage sludge.

Source apportionment of HMs in sewage sludge can control HM contamination through suggesting improvements in government policies and industrial processes; also can promote the utilization of sewage sludge in agricultural land as an effective inexpensive fertilizer.

## 4. Conclusions

Sewage sludge samples from 32 WWTPs in the Shanxi Province revealed that Zn, Cu, Cr, Pb, As, Hg and Cd content was within standard limits for land application and lower than other regions in China. The mean concentration of HMs was arranged in the following decreasing order: Zn > Cu > Cr > Pb > As > Hg > Cd. Based on HM pollution classes by *I*_geo_: Cu and Hg pollution were highest; Cd and Cr pollution were moderate; Zn, As and Pb pollution were lower. *I*_geo_ indicated that HM pollution in sewage sludge were uncontaminated to moderately contaminated. The correlation coefficient between Cu and Hg (*r* = 0.725), Cu and Pb (*r* = 0.658) as well as Hg and Pb (*r* = 0.648) was significant (*p* < 0.01). Zn and As (*r* = 0.467), Zn and Cd (*r* = 0.491) and As and Cd (*r* = 0.511) also had highly significant relationships (*p* < 0.01). The highly significant correlation among these HMs meant that they might have the same sources.

Sources of HM contamination in sewage sludge were identified as three components by PCA. The dominant source, accounting for 35.7% of the total variance, included Cu, Hg and Pb. These were classified as metallurgy, coking industry and traffic sources. The second source, accounting for 29.0% of the total variance, included Zn, As and Cd. This one was defined as household and water supply system sources. The last source, accounting for 16.2% of the total variance, had strong positive loading only on Cr, which correlated with no other metals. It was associated with special industry sources such as the leather tanning industry, textile manufacturing and chemical engineering.

Land use and landfill of sewage sludge are the main disposal methods in Shanxi, where land use accounts for 52.9% and landfills account for 37.3%. This will lead to potential increases in HMs in soils [14,15]. In order to mitigate this potential risk, proper policies on land application of sewage sludge should be formulated. Appropriate treatment technologies also should be adopted to reduce HM content in sewage sludge: the chemical extracting method efficiently removes Hg and Pb [52]; the electro kinetic technique removes Cu and Ni to a level of over 90% [53]; the bioleaching method removes Cr and Ni using acid thiobacillusferrooxidans extracted from activated sludge [54]. These treatments are therefore appropriate to promote safe use of sewage sludge.
